# 5′′-(2-Fluoro­benzyl­idene)-1′-(2-fluoro­phen­yl)-1′′-methyl-1′,2′,3′,5′,6′,7′,8′,8a’-octa­hydro­dispiro­[ace­naphthyl­ene-1,3′-indolizine-2′,3′′-piperidine]-2,4′′(1*H*)-dione

**DOI:** 10.1107/S1600536813019594

**Published:** 2013-07-20

**Authors:** R. Vishnupriya, J. Suresh, S. Sivakumar, R. Ranjith Kumar, P. L. Nilantha Lakshman

**Affiliations:** a Department of Physics, Madura College, Madurai 625 011, India; b Department of Organic Chemistry, School of Chemistry, Madurai Kamaraj University, Madurai 625 021, India; c Department of Food Science and Technology, University of Ruhuna, Mapalana, Kamburupitiya 81100, Sri Lanka

## Abstract

In the title compound, C_37_H_32_F_2_N_2_O_2_, the central six-membered piperidine ring adopts a twisted half-chair conformation, with the N and methyl­ene C atoms deviating by −0.2875 (16) and 0.4965 (15) Å, respectively, from the mean plane defined by the other four atoms. The piperidine connected to the octa­hydro­indolizine ring is in a half-chair conformation. The five-membered pyrrole ring adopts a slightly twisted envelope conformation with the piperidine C atom as the flap atom. The F and H atoms of both fluoro­benzene rings are disordered, with occupancy factors of 0.941 (3):0.059 (3) and 0.863 (3):0.137 (3). The mol­ecular structure features some intra­molecular C—H⋯O inter­actions. In the crystal, a supra­molecular zigzag chain sustained by C—H⋯F inter­actions parallel to the *c* axis is formed, generating a *C*(12) graph-set motif.

## Related literature

For indolizine derivatives, see: Medda *et al.* (2003 ▶). For background to spiro compounds, see: Caramella & Grunanger (1984 ▶); James *et al.* (1991 ▶); Kobayashi *et al.* (1991 ▶). For related structures, see: Sussman & Wodak (1973 ▶); Wodak (1975 ▶). For ring conformation analysis, see: Cremer & Pople (1975 ▶). For graph-set analysis of hydrogen bonds, see: Bernstein *et al.* (1995 ▶).
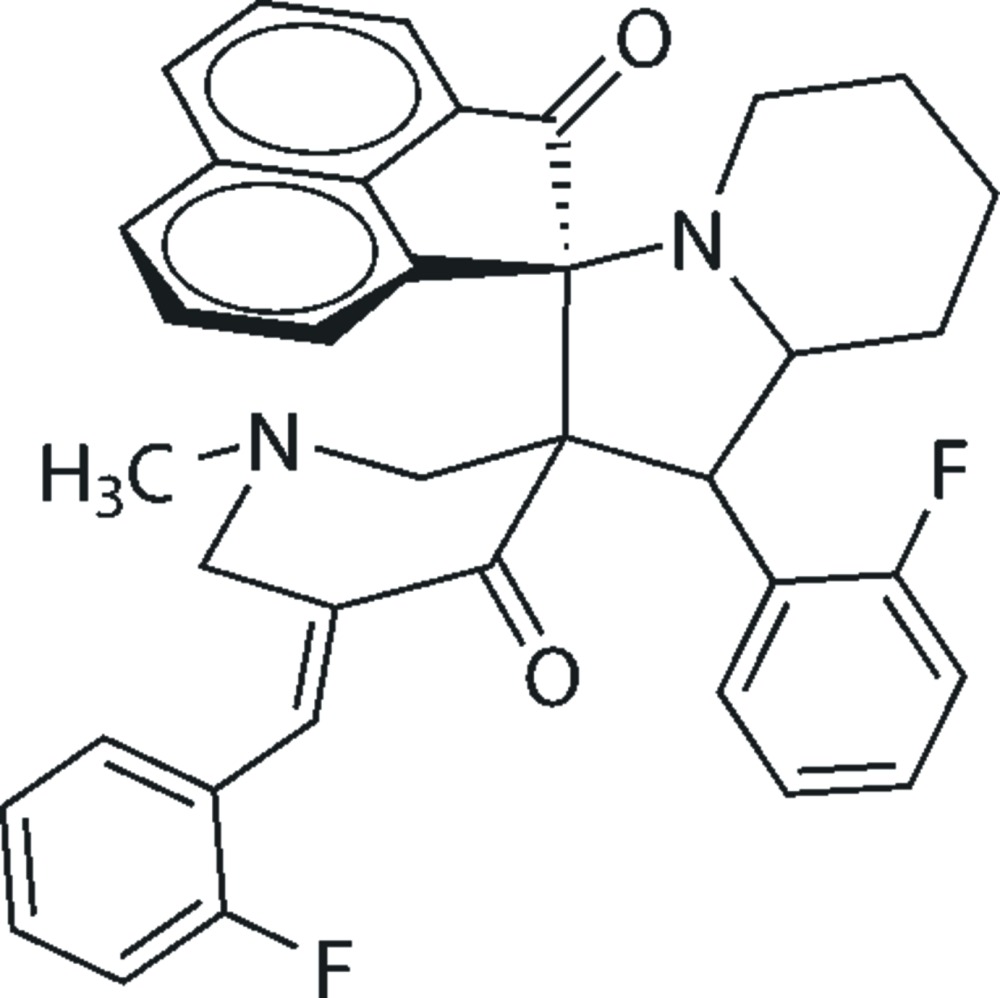


## Experimental

### 

#### Crystal data

C_37_H_32_F_2_N_2_O_2_
*M* *_r_* = 574.65
Monoclinic, 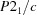

*a* = 8.5161 (3) Å
*b* = 16.8176 (6) Å
*c* = 20.5195 (6) Å
β = 99.845 (2)°
*V* = 2895.53 (17) Å^3^
*Z* = 4
Mo *K*α radiation
μ = 0.09 mm^−1^
*T* = 293 K
0.30 × 0.30 × 0.25 mm

#### Data collection

Bruker Kappa APEXII diffractometer
Absorption correction: multi-scan (*SADABS*; Sheldrick, 1996 ▶) *T*_min_ = 0.974, *T*_max_ = 0.978
27283 measured reflections
5702 independent reflections
4231 reflections with *I* > 2σ(*I*)
*R*_int_ = 0.033

#### Refinement

*R*[*F* ^2^ > 2σ(*F* ^2^)] = 0.052
*wR*(*F* ^2^) = 0.146
*S* = 1.02
5702 reflections
409 parameters
23 restraints
H-atom parameters constrained
Δρ_max_ = 0.70 e Å^−3^
Δρ_min_ = −0.45 e Å^−3^

### 

Data collection: *APEX2* (Bruker, 2004 ▶); cell refinement: *SAINT* (Bruker, 2004 ▶); data reduction: *SAINT*; program(s) used to solve structure: *SHELXS97* (Sheldrick, 2008 ▶); program(s) used to refine structure: *SHELXL97* (Sheldrick, 2008 ▶); molecular graphics: *PLATON* (Spek, 2009 ▶); software used to prepare material for publication: *SHELXL97*.

## Supplementary Material

Crystal structure: contains datablock(s) global, I. DOI: 10.1107/S1600536813019594/tk5239sup1.cif

Structure factors: contains datablock(s) I. DOI: 10.1107/S1600536813019594/tk5239Isup2.hkl

Additional supplementary materials:  crystallographic information; 3D view; checkCIF report

## Figures and Tables

**Table 1:** Hydrogen-bond geometry (Å, °)

*D*—H⋯*A*	*D*—H	H⋯*A*	*D*⋯*A*	*D*—H⋯*A*
C6—H6*A*⋯O2	0.97	2.35	2.930 (3)	118
C7—H7⋯O1	0.98	2.31	2.823 (2)	112
C22—H22⋯O1	0.93	2.58	3.146 (3)	120
C10—H10*B*⋯F1^i^	0.97	2.53	3.102 (3)	118

Symmetry code: (i) 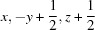.

**Fig. 1.**
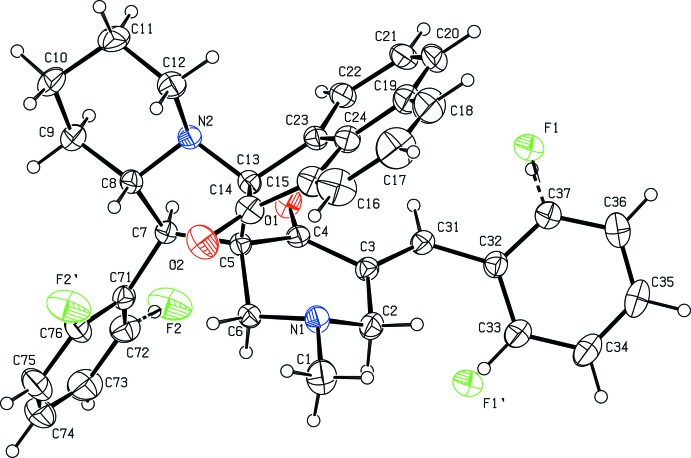
The molecular structure of (I), showing 30% probability displacement ellipsoids and the atom-numbering scheme.

**Fig. 2.**
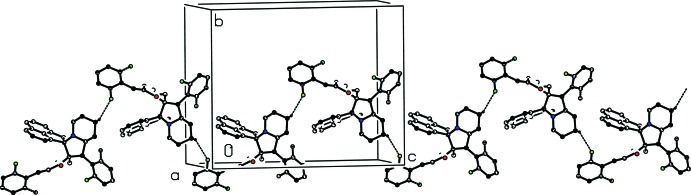
Partial packing diagram showing C11(12) motif.

## supplementary crystallographic information

### Comment

Spiro-compounds represent an important class of naturally occurring substances which in many cases exhibit important biological properties (Kobayashi *et al.*, 1991; James *et al.*, 1991). 1,3-Dipolar cycloaddition reactions are widely used for the construction of spiro-compounds (Caramella & Grunanger, 1984). It is also pertinent to note that the synthesis of biologically active indolizine derivatives continues to attract the attention of organic chemists because of their wide spectrum of biological activities (Medda *et al.*, 2003). Owing to their biological importance, we have synthesized and report here the crystal structure of the title indolizine compound.

In the title compound (Fig. 1), the six-membered piperidine ring adopts a twisted half-chair conformation, as evidenced by the puckering parameters q_2_ = 0.5457 (19) Å, θ = 35.2 (2)°, φ = 42.0 (4)° (Cremer & Pople, 1975). The sum of the bond angles around N1 [331.80 (1)°] indicates a pyramidal geometry. In the octahydroindolizine ring, the piperidine ring has a half-chair conformation and the pyrrole ring is in a slightly twisted envelope conformation, with the C8 atom at the flap of the envelope. The twist of the 2-fluorobenzene ring (C32–C37) with respect to the spiro junction is indicated by the torsion angle C3—C31—C32—C33 = 41.43 (2)°. The C8—N2 bond length [1.453 (2) Å] is comparable with C*sp*^2^—N*sp*^2^ distances found in similar structures (Sussman & Wodak, 1973; Wodak, 1975). The dihedral angle between the fluorobenzene rings is 74.56 (1)°, and these rings make angles of 36.91 (1) and 70.91 (1)° with the acenaphthene group. The molecular structure features some intramolecular C—H···O interactions.

In the crystal structure, a zigzag supramolecular chain sustained by a C—H···F interaction, generating a graph-set motif of C_1_^1^(12), parallel to the *c* axis, is formed (Fig. 2).

### Experimental

A mixture of 1-methyl-3,5-bis[(*E*)-2-fluorophenylmethylidene]tetrahydro-4(1*H*)-pyridinone (1 mmol), acenaphthenequinone (1 mmol) and piperidine-2-carboxylic acid (1 mmol) was dissolved in isopropyl alcohol (15 ml) and heated to reflux for 60 min. After completion of the reaction as evident from TLC, the mixture was poured into water (50 ml). The precipitated solid was filtered and washed with water (100 ml) to obtain a pure yellow solid having a melting point of 510 K (yield 92%)

### Refinement

H atoms were placed in calculated positions and allowed to ride on their carrier atoms, with C—H = 0.93–0.98 Å, and with *U*_iso_ = 1.2*U*_eq_(C) for CH_2_ and CH groups and *U*_iso_ = 1.5*U*_eq_(C) for the CH_3_ group. The two F and two H atoms of the fluorobenzene rings are disordered over two sets of sites in the ratios 0.941 (3):0.059 (3) and 0.863 (3):0.137 (3), respectively. The F atoms were refined as two sets of atoms (F1, F1', and F2, F2') sharing the same site [their xyz and U^ij^ parameters were equated by dummy atom constraints using the EADP command]. Also, FLAT and DFIX restraints were used to stabilize the refinement of the disordered atoms.

### Crystal data

**Table:**

C_37_H_32_F_2_N_2_O_2_	*F*(000) = 1208
*M**_r_* = 574.65	*D*_x_ = 1.318 Mg m^−^^3^
Monoclinic, *P*2_1_/*c*	Mo *K*α radiation, λ = 0.71073 Å
Hall symbol: -P 2ybc	Cell parameters from 2000 reflections
*a* = 8.5161 (3) Å	θ = 2–26°
*b* = 16.8176 (6) Å	µ = 0.09 mm^−^^1^
*c* = 20.5195 (6) Å	*T* = 293 K
β = 99.845 (2)°	Block, yellow
*V* = 2895.53 (17) Å^3^	0.30 × 0.30 × 0.25 mm
*Z* = 4	

### Data collection

**Table:**

Bruker Kappa APEXII diffractometer	5702 independent reflections
Radiation source: fine-focus sealed tube	4231 reflections with *I* > 2σ(*I*)
Graphite monochromator	*R*_int_ = 0.033
Detector resolution: 0 pixels mm^-1^	θ_max_ = 26.0°, θ_min_ = 2.4°
ω and φ scans	*h* = −10→10
Absorption correction: multi-scan (*SADABS*; Sheldrick, 1996)	*k* = −20→20
*T*_min_ = 0.974, *T*_max_ = 0.978	*l* = −25→22
27283 measured reflections	

### Refinement

**Table:**

Refinement on *F*^2^	Primary atom site location: structure-invariant direct methods
Least-squares matrix: full	Secondary atom site location: difference Fourier map
*R*[*F*^2^ > 2σ(*F*^2^)] = 0.052	Hydrogen site location: inferred from neighbouring sites
*wR*(*F*^2^) = 0.146	H-atom parameters constrained
*S* = 1.02	*w* = 1/[σ^2^(*F*_o_^2^) + (0.0673*P*)^2^ + 1.2896*P*] where *P* = (*F*_o_^2^ + 2*F*_c_^2^)/3
5702 reflections	(Δ/σ)_max_ < 0.001
409 parameters	Δρ_max_ = 0.70 e Å^−^^3^
23 restraints	Δρ_min_ = −0.45 e Å^−^^3^

### Special details

**Table:**

Geometry. All e.s.d.'s (except the e.s.d. in the dihedral angle between two l.s. planes) are estimated using the full covariance matrix. The cell e.s.d.'s are taken into account individually in the estimation of e.s.d.'s in distances, angles and torsion angles; correlations between e.s.d.'s in cell parameters are only used when they are defined by crystal symmetry. An approximate (isotropic) treatment of cell e.s.d.'s is used for estimating e.s.d.'s involving l.s. planes.
Refinement. Refinement of *F*^2^ against ALL reflections. The weighted *R*-factor *wR* and goodness of fit *S* are based on *F*^2^, conventional *R*-factors *R* are based on *F*, with *F* set to zero for negative *F*^2^. The threshold expression of *F*^2^ > σ(*F*^2^) is used only for calculating *R*-factors(gt) *etc*. and is not relevant to the choice of reflections for refinement. *R*-factors based on *F*^2^ are statistically about twice as large as those based on *F*, and *R*-factors based on ALL data will be even larger.

### Fractional atomic coordinates and isotropic or equivalent isotropic displacement parameters (Å^2^)

**Table:**

	*x*	*y*	*z*	*U*_iso_*/*U*_eq_	Occ. (<1)
C1	0.6712 (3)	0.02064 (19)	0.28763 (12)	0.0678 (7)	
H1A	0.7148	0.0582	0.3211	0.102*	
H1B	0.7321	0.0218	0.2524	0.102*	
H1C	0.6754	−0.0318	0.3064	0.102*	
C6	0.4080 (2)	0.04033 (11)	0.31277 (9)	0.0372 (4)	
H6A	0.4646	0.0653	0.3526	0.045*	
H6B	0.3845	−0.0142	0.3232	0.045*	
C5	0.2531 (2)	0.08512 (10)	0.28843 (8)	0.0318 (4)	
C4	0.1667 (2)	0.04161 (10)	0.22699 (9)	0.0328 (4)	
C3	0.2679 (2)	0.00480 (11)	0.18251 (9)	0.0352 (4)	
C2	0.4399 (2)	−0.01283 (14)	0.20876 (10)	0.0465 (5)	
H2A	0.4496	−0.0670	0.2252	0.056*	
H2B	0.5009	−0.0085	0.1731	0.056*	
C7	0.1462 (2)	0.09501 (10)	0.34238 (8)	0.0331 (4)	
H7	0.0357	0.0872	0.3205	0.040*	
C8	0.1632 (2)	0.18227 (11)	0.36217 (9)	0.0371 (4)	
H8	0.2630	0.1903	0.3931	0.045*	
C9	0.0260 (3)	0.21723 (12)	0.39059 (11)	0.0500 (5)	
H9A	0.0228	0.1939	0.4336	0.060*	
H9B	−0.0737	0.2051	0.3618	0.060*	
C10	0.0457 (3)	0.30692 (13)	0.39748 (13)	0.0624 (6)	
H10A	−0.0474	0.3294	0.4120	0.075*	
H10B	0.1379	0.3188	0.4308	0.075*	
C11	0.0666 (3)	0.34456 (13)	0.33238 (12)	0.0570 (6)	
H11A	−0.0316	0.3393	0.3009	0.068*	
H11B	0.0888	0.4008	0.3391	0.068*	
C12	0.2009 (3)	0.30590 (12)	0.30461 (11)	0.0499 (5)	
H12A	0.2063	0.3278	0.2613	0.060*	
H12B	0.3015	0.3164	0.3334	0.060*	
C13	0.2847 (2)	0.17411 (11)	0.26733 (9)	0.0347 (4)	
C14	0.4631 (2)	0.19945 (12)	0.29021 (10)	0.0451 (5)	
C15	0.5233 (3)	0.23152 (13)	0.23229 (12)	0.0523 (5)	
C16	0.6665 (3)	0.26351 (17)	0.22479 (16)	0.0771 (8)	
H16	0.7519	0.2644	0.2597	0.093*	
C17	0.6809 (4)	0.29505 (19)	0.16283 (19)	0.0900 (10)	
H17	0.7781	0.3166	0.1571	0.108*	
C18	0.5589 (4)	0.29523 (16)	0.11125 (16)	0.0782 (9)	
H18	0.5735	0.3180	0.0714	0.094*	
C19	0.4094 (3)	0.26143 (13)	0.11645 (12)	0.0575 (6)	
C20	0.2719 (4)	0.25647 (14)	0.06846 (11)	0.0645 (7)	
H20	0.2735	0.2755	0.0260	0.077*	
C21	0.1360 (3)	0.22419 (14)	0.08309 (11)	0.0591 (6)	
H21	0.0466	0.2213	0.0501	0.071*	
C22	0.1254 (3)	0.19480 (12)	0.14682 (10)	0.0460 (5)	
H22	0.0295	0.1753	0.1562	0.055*	
C23	0.2581 (2)	0.19573 (11)	0.19405 (9)	0.0386 (4)	
C24	0.3982 (3)	0.22939 (12)	0.17870 (10)	0.0457 (5)	
C31	0.1995 (2)	−0.00713 (11)	0.11974 (9)	0.0383 (4)	
H31	0.0938	0.0090	0.1087	0.046*	
C32	0.2695 (2)	−0.04237 (11)	0.06619 (10)	0.0423 (5)	
C37	0.23416 (17)	−0.01148 (8)	0.00325 (11)	0.0520 (5)	
C36	0.2894 (3)	−0.04094 (16)	−0.05035 (12)	0.0742 (8)	
H36	0.2612	−0.0178	−0.0918	0.089*	
C35	0.3877 (3)	−0.10561 (18)	−0.04128 (14)	0.0871 (10)	
H35	0.4284	−0.1267	−0.0768	0.104*	
C34	0.4265 (3)	−0.13946 (17)	0.02006 (14)	0.0829 (9)	
H34	0.4929	−0.1837	0.0258	0.100*	
C33	0.3685 (3)	−0.10869 (14)	0.07303 (12)	0.0602 (6)	
C71	0.1764 (2)	0.03691 (11)	0.39982 (9)	0.0381 (4)	
C72	0.09500 (17)	−0.03280 (12)	0.39963 (9)	0.0517 (5)	
C73	0.1094 (3)	−0.08554 (14)	0.45142 (15)	0.0702 (8)	
H73	0.0499	−0.1322	0.4480	0.084*	
C74	0.2128 (4)	−0.06792 (17)	0.50795 (14)	0.0744 (8)	
H74	0.2247	−0.1025	0.5438	0.089*	
C75	0.2982 (3)	0.00060 (17)	0.51141 (12)	0.0733 (8)	
H75	0.3692	0.0129	0.5497	0.088*	
C76	0.2799 (3)	0.05192 (14)	0.45829 (11)	0.0573 (6)	
N2	0.17327 (19)	0.22003 (9)	0.29931 (7)	0.0376 (4)	
N1	0.50577 (18)	0.04147 (10)	0.26180 (8)	0.0425 (4)	
O1	0.02247 (15)	0.03953 (8)	0.21400 (7)	0.0444 (3)	
O2	0.53158 (19)	0.20027 (10)	0.34713 (8)	0.0602 (4)	
F1	0.13693 (19)	0.05231 (8)	−0.00574 (10)	0.0724 (5)	0.941 (3)
F1'	0.406 (2)	−0.1403 (13)	0.1341 (6)	0.0724 (5)	0.059 (3)
F2	−0.0043 (2)	−0.05342 (10)	0.34383 (8)	0.0805 (6)	0.863 (3)
F2'	0.3741 (8)	0.1167 (3)	0.4695 (7)	0.0805 (6)	0.137 (3)
H33	0.396 (3)	−0.1312 (18)	0.1148 (8)	0.097*	0.941 (3)
H33'	0.167 (3)	0.033 (3)	0.00 (4)	0.097*	0.059 (3)
H76	0.342 (2)	0.0977 (9)	0.464 (2)	0.097*	0.863 (3)
H76'	0.0232 (6)	−0.0475 (9)	0.3622 (4)	0.097*	0.137 (3)

### Atomic displacement parameters (Å^2^)

**Table:**

	*U*^11^	*U*^22^	*U*^33^	*U*^12^	*U*^13^	*U*^23^
C1	0.0343 (11)	0.110 (2)	0.0578 (14)	0.0101 (12)	0.0041 (10)	−0.0045 (14)
C6	0.0373 (10)	0.0413 (10)	0.0325 (9)	0.0031 (8)	0.0047 (8)	0.0015 (8)
C5	0.0337 (9)	0.0317 (9)	0.0302 (9)	−0.0012 (7)	0.0065 (7)	0.0000 (7)
C4	0.0344 (10)	0.0285 (9)	0.0354 (10)	0.0002 (7)	0.0060 (8)	0.0005 (7)
C3	0.0379 (10)	0.0314 (9)	0.0370 (10)	−0.0001 (7)	0.0087 (8)	−0.0013 (8)
C2	0.0428 (11)	0.0563 (13)	0.0413 (11)	0.0096 (9)	0.0100 (9)	−0.0044 (9)
C7	0.0348 (9)	0.0324 (9)	0.0321 (9)	0.0007 (7)	0.0060 (7)	−0.0002 (7)
C8	0.0457 (10)	0.0335 (10)	0.0317 (9)	0.0014 (8)	0.0050 (8)	−0.0014 (7)
C9	0.0616 (13)	0.0432 (12)	0.0475 (12)	0.0096 (10)	0.0160 (10)	−0.0018 (9)
C10	0.0838 (17)	0.0432 (13)	0.0642 (15)	0.0152 (12)	0.0241 (13)	−0.0094 (11)
C11	0.0725 (15)	0.0312 (11)	0.0649 (15)	0.0061 (10)	0.0052 (12)	−0.0046 (10)
C12	0.0671 (14)	0.0310 (10)	0.0508 (12)	−0.0042 (9)	0.0080 (11)	−0.0013 (9)
C13	0.0385 (10)	0.0328 (9)	0.0320 (9)	−0.0037 (7)	0.0041 (8)	0.0000 (7)
C14	0.0441 (11)	0.0422 (11)	0.0473 (12)	−0.0094 (9)	0.0028 (9)	−0.0016 (9)
C15	0.0526 (13)	0.0447 (12)	0.0613 (14)	−0.0128 (10)	0.0145 (11)	0.0022 (10)
C16	0.0623 (16)	0.0731 (18)	0.098 (2)	−0.0264 (14)	0.0191 (15)	0.0128 (16)
C17	0.081 (2)	0.081 (2)	0.118 (3)	−0.0280 (17)	0.047 (2)	0.0140 (19)
C18	0.103 (2)	0.0587 (16)	0.087 (2)	−0.0046 (15)	0.0547 (19)	0.0182 (15)
C19	0.0839 (17)	0.0402 (12)	0.0560 (14)	0.0020 (11)	0.0331 (13)	0.0073 (10)
C20	0.107 (2)	0.0498 (14)	0.0410 (13)	0.0132 (14)	0.0231 (14)	0.0126 (10)
C21	0.0861 (18)	0.0485 (13)	0.0390 (12)	0.0135 (12)	0.0005 (12)	0.0052 (10)
C22	0.0575 (13)	0.0395 (11)	0.0387 (11)	0.0036 (9)	0.0016 (9)	0.0021 (8)
C23	0.0522 (11)	0.0290 (9)	0.0347 (10)	−0.0008 (8)	0.0077 (9)	0.0012 (7)
C24	0.0591 (13)	0.0338 (10)	0.0474 (12)	−0.0035 (9)	0.0185 (10)	0.0026 (9)
C31	0.0406 (10)	0.0352 (10)	0.0393 (10)	0.0022 (8)	0.0073 (8)	−0.0025 (8)
C32	0.0455 (11)	0.0430 (11)	0.0387 (10)	0.0012 (8)	0.0078 (9)	−0.0056 (8)
C37	0.0636 (14)	0.0479 (13)	0.0453 (12)	0.0069 (10)	0.0121 (11)	−0.0007 (10)
C36	0.100 (2)	0.082 (2)	0.0441 (14)	0.0115 (16)	0.0233 (14)	−0.0010 (13)
C35	0.110 (2)	0.097 (2)	0.0615 (17)	0.0279 (19)	0.0363 (17)	−0.0179 (16)
C34	0.096 (2)	0.087 (2)	0.0681 (18)	0.0404 (17)	0.0198 (16)	−0.0181 (15)
C33	0.0709 (16)	0.0585 (15)	0.0510 (13)	0.0196 (12)	0.0096 (12)	−0.0076 (11)
C71	0.0424 (10)	0.0361 (10)	0.0395 (10)	0.0038 (8)	0.0173 (8)	0.0028 (8)
C72	0.0491 (12)	0.0453 (12)	0.0626 (14)	−0.0007 (10)	0.0152 (11)	0.0046 (10)
C73	0.0805 (18)	0.0481 (14)	0.091 (2)	−0.0039 (12)	0.0401 (16)	0.0191 (13)
C74	0.096 (2)	0.0721 (18)	0.0621 (17)	0.0162 (16)	0.0322 (16)	0.0298 (14)
C75	0.097 (2)	0.0772 (19)	0.0443 (13)	0.0044 (16)	0.0085 (13)	0.0168 (13)
C76	0.0761 (16)	0.0577 (14)	0.0377 (11)	0.0021 (12)	0.0083 (11)	0.0093 (10)
N2	0.0485 (9)	0.0292 (8)	0.0350 (8)	−0.0001 (7)	0.0068 (7)	−0.0005 (6)
N1	0.0315 (8)	0.0581 (11)	0.0378 (9)	0.0023 (7)	0.0056 (7)	0.0001 (8)
O1	0.0343 (7)	0.0511 (8)	0.0475 (8)	−0.0042 (6)	0.0058 (6)	−0.0128 (6)
O2	0.0546 (9)	0.0696 (11)	0.0504 (9)	−0.0159 (8)	−0.0078 (7)	−0.0063 (8)
F1	0.1051 (13)	0.0591 (9)	0.0528 (9)	0.0272 (8)	0.0135 (9)	0.0110 (7)
F1'	0.1051 (13)	0.0591 (9)	0.0528 (9)	0.0272 (8)	0.0135 (9)	0.0110 (7)
F2	0.0798 (12)	0.0546 (10)	0.0961 (13)	−0.0212 (8)	−0.0158 (10)	0.0144 (9)
F2'	0.0798 (12)	0.0546 (10)	0.0961 (13)	−0.0212 (8)	−0.0158 (10)	0.0144 (9)

### Geometric parameters (Å, º)

**Table:**

C1—N1	1.460 (3)	C17—C18	1.350 (4)
C1—H1A	0.9600	C17—H17	0.9300
C1—H1B	0.9600	C18—C19	1.416 (4)
C1—H1C	0.9600	C18—H18	0.9300
C6—N1	1.444 (2)	C19—C20	1.398 (4)
C6—C5	1.527 (2)	C19—C24	1.405 (3)
C6—H6A	0.9700	C20—C21	1.358 (4)
C6—H6B	0.9700	C20—H20	0.9300
C5—C4	1.533 (2)	C21—C22	1.415 (3)
C5—C7	1.558 (2)	C21—H21	0.9300
C5—C13	1.594 (2)	C22—C23	1.357 (3)
C4—O1	1.212 (2)	C22—H22	0.9300
C4—C3	1.492 (2)	C23—C24	1.404 (3)
C3—C31	1.335 (3)	C31—C32	1.463 (3)
C3—C2	1.501 (3)	C31—H31	0.9300
C2—N1	1.457 (3)	C32—C37	1.377 (3)
C2—H2A	0.9700	C32—C33	1.390 (3)
C2—H2B	0.9700	C37—F1	1.3483 (10)
C7—C71	1.519 (2)	C37—C36	1.361 (3)
C7—C8	1.523 (2)	C37—H33'	0.930 (2)
C7—H7	0.9800	C36—C35	1.366 (4)
C8—N2	1.453 (2)	C36—H36	0.9300
C8—C9	1.511 (3)	C35—C34	1.369 (4)
C8—H8	0.9800	C35—H35	0.9300
C9—C10	1.522 (3)	C34—C33	1.370 (3)
C9—H9A	0.9700	C34—H34	0.9300
C9—H9B	0.9700	C33—F1'	1.3494 (11)
C10—C11	1.516 (3)	C33—H33	0.9298 (11)
C10—H10A	0.9700	C71—C72	1.362 (3)
C10—H10B	0.9700	C71—C76	1.385 (3)
C11—C12	1.510 (3)	C72—F2	1.3471 (10)
C11—H11A	0.9700	C72—C73	1.374 (3)
C11—H11B	0.9700	C72—H76'	0.9300 (11)
C12—N2	1.464 (2)	C73—C74	1.363 (4)
C12—H12A	0.9700	C73—H73	0.9300
C12—H12B	0.9700	C74—C75	1.358 (4)
C13—N2	1.463 (2)	C74—H74	0.9300
C13—C23	1.526 (3)	C75—C76	1.378 (3)
C13—C14	1.570 (3)	C75—H75	0.9300
C14—O2	1.213 (2)	C76—F2'	1.3485 (11)
C14—C15	1.475 (3)	C76—H76	0.9298 (11)
C15—C16	1.365 (3)	F1—H33'	0.42 (15)
C15—C24	1.395 (3)	F1'—H33	0.420 (4)
C16—C17	1.402 (4)	F2—H76'	0.420 (2)
C16—H16	0.9300	F2'—H76	0.423 (10)
			
N1—C1—H1A	109.5	C16—C17—H17	118.8
N1—C1—H1B	109.5	C17—C18—C19	121.4 (3)
H1A—C1—H1B	109.5	C17—C18—H18	119.3
N1—C1—H1C	109.5	C19—C18—H18	119.3
H1A—C1—H1C	109.5	C20—C19—C24	116.0 (2)
H1B—C1—H1C	109.5	C20—C19—C18	128.8 (2)
N1—C6—C5	109.23 (15)	C24—C19—C18	115.1 (3)
N1—C6—H6A	109.8	C21—C20—C19	120.7 (2)
C5—C6—H6A	109.8	C21—C20—H20	119.6
N1—C6—H6B	109.8	C19—C20—H20	119.6
C5—C6—H6B	109.8	C20—C21—C22	122.4 (2)
H6A—C6—H6B	108.3	C20—C21—H21	118.8
C6—C5—C4	107.45 (14)	C22—C21—H21	118.8
C6—C5—C7	113.42 (14)	C23—C22—C21	118.6 (2)
C4—C5—C7	112.26 (14)	C23—C22—H22	120.7
C6—C5—C13	112.05 (14)	C21—C22—H22	120.7
C4—C5—C13	107.73 (13)	C22—C23—C24	118.69 (18)
C7—C5—C13	103.84 (14)	C22—C23—C13	131.77 (19)
O1—C4—C3	121.52 (16)	C24—C23—C13	109.18 (17)
O1—C4—C5	121.37 (16)	C15—C24—C23	113.42 (18)
C3—C4—C5	117.06 (15)	C15—C24—C19	123.1 (2)
C31—C3—C4	116.74 (17)	C23—C24—C19	123.4 (2)
C31—C3—C2	123.69 (17)	C3—C31—C32	128.23 (18)
C4—C3—C2	119.49 (16)	C3—C31—H31	115.9
N1—C2—C3	111.81 (16)	C32—C31—H31	115.9
N1—C2—H2A	109.3	C37—C32—C33	115.40 (18)
C3—C2—H2A	109.3	C37—C32—C31	119.99 (17)
N1—C2—H2B	109.3	C33—C32—C31	124.57 (19)
C3—C2—H2B	109.3	F1—C37—C36	117.9 (2)
H2A—C2—H2B	107.9	F1—C37—C32	117.34 (18)
C71—C7—C8	114.66 (15)	C36—C37—C32	124.74 (18)
C71—C7—C5	116.15 (14)	F1—C37—H33'	4 (10)
C8—C7—C5	104.59 (14)	C36—C37—H33'	121 (10)
C71—C7—H7	107.0	C32—C37—H33'	114 (10)
C8—C7—H7	107.0	C37—C36—C35	117.9 (2)
C5—C7—H7	107.0	C37—C36—H36	121.0
N2—C8—C9	109.84 (16)	C35—C36—H36	121.0
N2—C8—C7	101.64 (14)	C36—C35—C34	120.1 (2)
C9—C8—C7	115.67 (17)	C36—C35—H35	119.9
N2—C8—H8	109.8	C34—C35—H35	119.9
C9—C8—H8	109.8	C35—C34—C33	120.6 (3)
C7—C8—H8	109.8	C35—C34—H34	119.7
C8—C9—C10	109.95 (19)	C33—C34—H34	119.7
C8—C9—H9A	109.7	F1'—C33—C34	121.8 (13)
C10—C9—H9A	109.7	F1'—C33—C32	117.0 (13)
C8—C9—H9B	109.7	C34—C33—C32	121.2 (2)
C10—C9—H9B	109.7	F1'—C33—H33	1 (4)
H9A—C9—H9B	108.2	C34—C33—H33	121 (3)
C11—C10—C9	111.09 (18)	C32—C33—H33	118 (3)
C11—C10—H10A	109.4	C72—C71—C76	114.05 (18)
C9—C10—H10A	109.4	C72—C71—C7	122.22 (17)
C11—C10—H10B	109.4	C76—C71—C7	123.59 (18)
C9—C10—H10B	109.4	F2—C72—C71	117.99 (18)
H10A—C10—H10B	108.0	F2—C72—C73	116.8 (2)
C12—C11—C10	111.32 (18)	C71—C72—C73	125.16 (19)
C12—C11—H11A	109.4	F2—C72—H76'	2.4 (8)
C10—C11—H11A	109.4	C71—C72—H76'	119.5 (11)
C12—C11—H11B	109.4	C73—C72—H76'	115.3 (11)
C10—C11—H11B	109.4	C74—C73—C72	118.5 (2)
H11A—C11—H11B	108.0	C74—C73—H73	120.8
N2—C12—C11	109.28 (18)	C72—C73—H73	120.8
N2—C12—H12A	109.8	C75—C74—C73	119.4 (2)
C11—C12—H12A	109.8	C75—C74—H74	120.3
N2—C12—H12B	109.8	C73—C74—H74	120.3
C11—C12—H12B	109.8	C74—C75—C76	120.2 (3)
H12A—C12—H12B	108.3	C74—C75—H75	119.9
N2—C13—C23	108.52 (15)	C76—C75—H75	119.9
N2—C13—C14	113.04 (15)	F2'—C76—C75	112.8 (7)
C23—C13—C14	101.71 (15)	F2'—C76—C71	124.5 (7)
N2—C13—C5	102.78 (14)	C75—C76—C71	122.7 (2)
C23—C13—C5	119.21 (14)	F2'—C76—H76	3 (4)
C14—C13—C5	111.93 (15)	C75—C76—H76	116 (3)
O2—C14—C15	126.59 (19)	C71—C76—H76	122 (3)
O2—C14—C13	124.88 (19)	C8—N2—C13	107.98 (14)
C15—C14—C13	108.06 (17)	C8—N2—C12	113.46 (15)
C16—C15—C24	119.9 (2)	C13—N2—C12	116.45 (16)
C16—C15—C14	132.5 (2)	C6—N1—C2	109.67 (15)
C24—C15—C14	107.59 (18)	C6—N1—C1	111.95 (16)
C15—C16—C17	118.0 (3)	C2—N1—C1	110.18 (17)
C15—C16—H16	121.0	C37—F1—H33'	8 (10)
C17—C16—H16	121.0	C33—F1'—H33	3 (9)
C18—C17—C16	122.4 (3)	C72—F2—H76'	5.3 (17)
C18—C17—H17	118.8	C76—F2'—H76	7 (8)
			
N1—C6—C5—C4	61.26 (19)	C5—C13—C23—C24	−124.67 (18)
N1—C6—C5—C7	−174.07 (15)	C16—C15—C24—C23	−179.3 (2)
N1—C6—C5—C13	−56.89 (19)	C14—C15—C24—C23	−2.3 (3)
C6—C5—C4—O1	147.63 (17)	C16—C15—C24—C19	−2.2 (4)
C7—C5—C4—O1	22.3 (2)	C14—C15—C24—C19	174.7 (2)
C13—C5—C4—O1	−91.5 (2)	C22—C23—C24—C15	176.11 (19)
C6—C5—C4—C3	−34.8 (2)	C13—C23—C24—C15	2.2 (2)
C7—C5—C4—C3	−160.15 (15)	C22—C23—C24—C19	−0.9 (3)
C13—C5—C4—C3	86.13 (18)	C13—C23—C24—C19	−174.83 (19)
O1—C4—C3—C31	21.0 (3)	C20—C19—C24—C15	−178.3 (2)
C5—C4—C3—C31	−156.56 (17)	C18—C19—C24—C15	1.1 (3)
O1—C4—C3—C2	−162.03 (18)	C20—C19—C24—C23	−1.5 (3)
C5—C4—C3—C2	20.4 (2)	C18—C19—C24—C23	177.8 (2)
C31—C3—C2—N1	148.31 (19)	C4—C3—C31—C32	−179.44 (17)
C4—C3—C2—N1	−28.4 (3)	C2—C3—C31—C32	3.7 (3)
C6—C5—C7—C71	−21.1 (2)	C3—C31—C32—C37	−140.96 (19)
C4—C5—C7—C71	100.96 (18)	C3—C31—C32—C33	41.4 (3)
C13—C5—C7—C71	−142.95 (15)	C33—C32—C37—F1	179.21 (15)
C6—C5—C7—C8	106.39 (17)	C31—C32—C37—F1	1.39 (19)
C4—C5—C7—C8	−131.58 (15)	C33—C32—C37—C36	−0.40 (12)
C13—C5—C7—C8	−15.49 (17)	C31—C32—C37—C36	−178.22 (17)
C71—C7—C8—N2	164.53 (14)	F1—C37—C36—C35	−179.91 (17)
C5—C7—C8—N2	36.16 (17)	C32—C37—C36—C35	−0.30 (14)
C71—C7—C8—C9	−76.6 (2)	C37—C36—C35—C34	0.7 (3)
C5—C7—C8—C9	155.07 (16)	C36—C35—C34—C33	−0.5 (4)
N2—C8—C9—C10	−56.4 (2)	C35—C34—C33—F1'	−179.2 (3)
C7—C8—C9—C10	−170.73 (18)	C35—C34—C33—C32	−0.3 (4)
C8—C9—C10—C11	54.0 (3)	C37—C32—C33—F1'	179.7 (2)
C9—C10—C11—C12	−53.7 (3)	C31—C32—C33—F1'	−2.6 (4)
C10—C11—C12—N2	54.7 (3)	C37—C32—C33—C34	0.7 (3)
C6—C5—C13—N2	−133.40 (15)	C31—C32—C33—C34	178.4 (2)
C4—C5—C13—N2	108.61 (15)	C8—C7—C71—C72	145.43 (14)
C7—C5—C13—N2	−10.62 (17)	C5—C7—C71—C72	−92.28 (17)
C6—C5—C13—C23	106.60 (18)	C8—C7—C71—C76	−30.1 (2)
C4—C5—C13—C23	−11.4 (2)	C5—C7—C71—C76	92.18 (19)
C7—C5—C13—C23	−130.62 (17)	C76—C71—C72—F2	−177.74 (15)
C6—C5—C13—C14	−11.8 (2)	C7—C71—C72—F2	6.32 (17)
C4—C5—C13—C14	−129.79 (16)	C76—C71—C72—C73	0.17 (11)
C7—C5—C13—C14	110.98 (16)	C7—C71—C72—C73	−175.77 (15)
N2—C13—C14—O2	56.2 (3)	F2—C72—C73—C74	177.86 (17)
C23—C13—C14—O2	172.4 (2)	C71—C72—C73—C74	−0.07 (13)
C5—C13—C14—O2	−59.3 (3)	C72—C73—C74—C75	−0.1 (3)
N2—C13—C14—C15	−116.36 (18)	C73—C74—C75—C76	0.2 (3)
C23—C13—C14—C15	−0.2 (2)	C74—C75—C76—F2'	−179.5 (3)
C5—C13—C14—C15	128.16 (17)	C74—C75—C76—C71	−0.1 (3)
O2—C14—C15—C16	5.5 (4)	C72—C71—C76—F2'	179.2 (2)
C13—C14—C15—C16	177.9 (3)	C7—C71—C76—F2'	−4.9 (4)
O2—C14—C15—C24	−170.9 (2)	C72—C71—C76—C75	−0.1 (2)
C13—C14—C15—C24	1.4 (2)	C7—C71—C76—C75	175.81 (19)
C24—C15—C16—C17	1.4 (4)	C9—C8—N2—C13	−168.66 (16)
C14—C15—C16—C17	−174.7 (3)	C7—C8—N2—C13	−45.67 (18)
C15—C16—C17—C18	0.5 (5)	C9—C8—N2—C12	60.7 (2)
C16—C17—C18—C19	−1.6 (5)	C7—C8—N2—C12	−176.30 (16)
C17—C18—C19—C20	−179.9 (3)	C23—C13—N2—C8	162.33 (15)
C17—C18—C19—C24	0.8 (4)	C14—C13—N2—C8	−85.64 (18)
C24—C19—C20—C21	1.7 (3)	C5—C13—N2—C8	35.19 (17)
C18—C19—C20—C21	−177.6 (3)	C23—C13—N2—C12	−68.7 (2)
C19—C20—C21—C22	0.5 (4)	C14—C13—N2—C12	43.3 (2)
C20—C21—C22—C23	−3.0 (3)	C5—C13—N2—C12	164.16 (15)
C21—C22—C23—C24	3.1 (3)	C11—C12—N2—C8	−59.3 (2)
C21—C22—C23—C13	175.42 (19)	C11—C12—N2—C13	174.40 (16)
N2—C13—C23—C22	−54.5 (3)	C5—C6—N1—C2	−73.89 (19)
C14—C13—C23—C22	−173.9 (2)	C5—C6—N1—C1	163.50 (19)
C5—C13—C23—C22	62.5 (3)	C3—C2—N1—C6	54.3 (2)
N2—C13—C23—C24	118.30 (17)	C3—C2—N1—C1	177.93 (18)
C14—C13—C23—C24	−1.1 (2)		

### Hydrogen-bond geometry (Å, º)

**Table:**

*D*—H···*A*	*D*—H	H···*A*	*D*···*A*	*D*—H···*A*
C6—H6*A*···O2	0.97	2.35	2.930 (3)	118
C7—H7···O1	0.98	2.31	2.823 (2)	112
C22—H22···O1	0.93	2.58	3.146 (3)	120
C10—H10*B*···F1^i^	0.97	2.53	3.102 (3)	118

Symmetry code: (i) *x*, −*y*+1/2, *z*+1/2.
